# Secondary and Tertiary Transmission of Vaccinia Virus from US Military Service Member

**DOI:** 10.3201/eid1704.101316

**Published:** 2011-04

**Authors:** Gregory E. Young, Christina M. Hidalgo, Ann Sullivan-Frohm, Cynthia Schulte, Stephen Davis, Cassandra Kelly-Cirino, Christina Egan, Kimberly Wilkins, Ginny L. Emerson, Kimberly Noyes, Debra Blog

**Affiliations:** Author affiliations: New York State Department of Health, Buffalo, New York, USA (G.E. Young, C.M. Hidalgo, A. Sullivan-Frohm);; New York State Department of Health, Albany, New York, USA (C. Schulte, S. Davis, C. Kelly-Cirino, C. Egan, K. Noyes, D. Blog);; Centers for Disease Control and Prevention, Atlanta, Georgia, USA (K. Wilkins, G.L. Emerson)

**Keywords:** Military vaccination, secondary transmission, tertiary transmission, vaccinia, smallpox vaccination, wrestling, viruses, contact transmission, dispatch

## Abstract

During February and March 2010, the New York State Department of Health investigated secondary and tertiary vaccinia contact transmission from a military vaccinee to 4 close contacts. Identification of these cases underscores the need for strict adherence to postvaccination infection control guidance to avoid transmission of the live virus.

Vaccinia virus (VACV) is the live viral component of smallpox vaccine. Exposure to the vaccination site can result in contact transmission or inadvertent autoinoculation, which often is self-limited (*1**,**2*). However, severe complications can occur, especially in persons with underlying risk factors (e.g., immunodeficiencies, atopic dermatitis, or pregnancy) (*1*). During December 2002–May 2009, the reported rate of contact transmission for US military personnel was 5 cases per 100,000 persons; intimate and sports-related contact were the most commonly cited risks (*3*).

On March 11, 2010, the New York State Department of Health (NYSDOH) was notified of a suspected case of vaccinia in a person who had been exposed to a military service member recently vaccinated against smallpox. NYSDOH and the Centers for Disease Control and Prevention (CDC) conducted an investigation to identify the source of infection and potential contacts. One additional case of contact transmission from the primary vaccinee and 2 cases of tertiary transmission were confirmed. This investigation underscores the need for strict adherence to postvaccination infection control guidance to avoid transmission of the live virus.

## The Study

**Figure 1:**
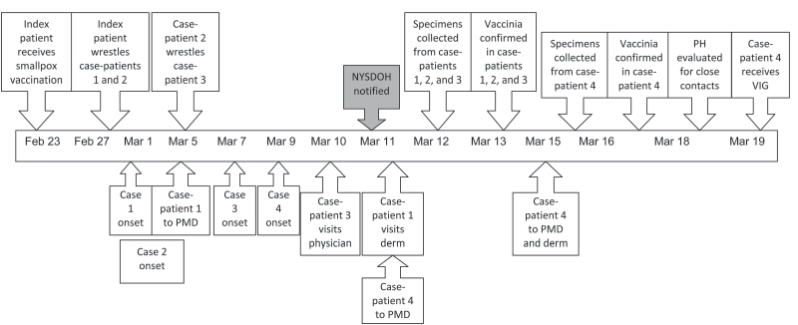
Timeline of investigation of secondary and tertiary transmission of vaccinia virus from a US military service member, New York, USA, 2010. NYSDOH, New York State Department of Health; PH, public health; VIG, varicella immune globulin; PMD, private physician; derm, dermatologist.

On February 23, 2010, a military service member preparing for deployment received smallpox vaccination and was counseled by the US Department of Defense about postvaccination care and infection control. On February 27, the index patient wrestled 2 persons in a semiprofessional match, during which the dressing covering the vaccination site was detached. Within 3 days, skin lesions developed in both contacts. One of these 2 wrestlers participated in another wrestling match on March 5, exposing a third person in whom lesions on the chest developed. A fourth contact, a household member of a wrestler from the February 27 match, had lesions develop on the face. Test results for all 4 persons were positive for VACV (Table). Each case was followed through resolution of lesions. Skin lesions developed in 3 additional contacts. All were examined, and test results of specimens were negative for VACV (Figure 1).

**Table:** Cases of laboratory-confirmed secondary and tertiary transmission of vaccinia virus from US military service member, New York, USA, 2010*

No.†	Transmission type	Age, y/sex	Exposure				Illness		Underlying risk factors	VIG	Duration of lesions, wk
			Date	Source	Type		Onset	Location of lesions			
1	Secondary	26/M	Feb 27	Military vaccinee	Wrestling		Mar 1	Face, neck, chest	None	No	≈3
2	Secondary	24/M	Feb 27	Military vaccinee	Wrestling		≈Mar 1	Face, neck, chin, eye	None	No‡	≈3
3	Tertiary	25/M	Mar 5	Case-patient 1	Wrestling		Mar 7	Chest, trunk, arm	Mild eczema	No	≈7
4	Tertiary	29/F	After March	Case-patient 1	Household		Mar 9	Face, mandible, nostril	None	Yes	≈3

*VIG, varicella immune globulin. †Case-patient no. ‡Use of VIG not indicated; treated with trifluridine ophthalmic solution (*3*).

### Case-Patient 1

**Figure 2:**
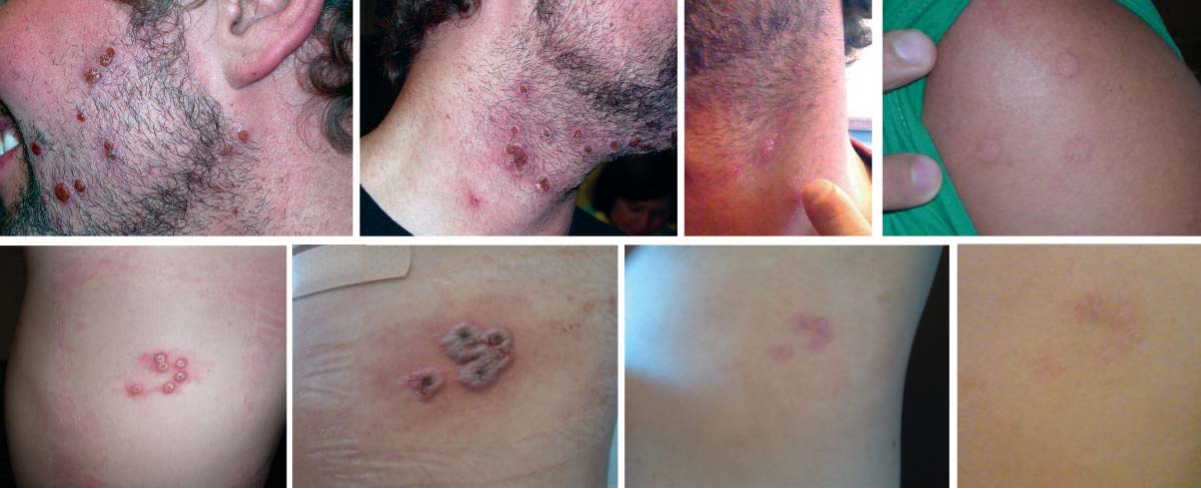
Vaccinia lesions in patients with secondary and tertiary cases, New York, USA, 2010. Top row, case-patient 1; bottom row, case-patient 3.

On March 11, a 26-year-old male wrestler with no noteworthy medical history visited a dermatologist after being referred by his private physician who had prescribed trimethoprim/sulfamethoxazole for presumed methicillin-resistant *Staphylococcus aureus* from lesions on his face, neck, and chest (Figure 2). The lesions developed suddenly, starting March 1, two days after wrestling. Molluscum contagiosum and impetigo were included in the differential diagnosis, and a culture was sent to a local laboratory to test for methicillin-resistant *S*. *aureus*. The dermatologist notified the local health department of the patient’s possible exposure to VACV. A NYSDOH public health physician evaluated the patient on March 12. On examination, he had clinically compatible VACV lesions, including papular, umbilicated lesions with overlying vesicles and a few pustules.

### Case-Patient 2

On March 11, a 24-year-old male wrestler with no noteworthy medical history was contacted by the NYSDOH because he was a wrestling contact of the vaccinee. He reported lesions on his face, neck, chin, and left eye that developed several days after the wrestling event and were associated with substantial pruritus, exudate, and erythema. At his public health evaluation on March 12, he had numerous papular lesions that were umbilicated with overlying vesicles, several of which were draining serous fluid, and VACV was clinically diagnosed. He had involvement of the left lower eyelid with substantial erythema and early blepharitis. He received slit-lamp examination by a local ophthalmologist, who after consultation with CDC and NYSDOH, treated him with trifluridine ophthalmic solution (*4*). The blepharitis and eyelid erythema resolved within 48 hours after initiation of trifluridine.

### Case-Patient 3

On March 10, a 25-year-old male wrestler and roommate of the vaccinee sought treatment for vesicular lesions on his trunk and chest. His medical history included recent mild eczema involving his hands. He reported that several pruritic papules had developed 2 days after wrestling case-patient 1. The physician thought the lesions appeared to be molluscum contagiosum, but given the patient’s history, consulted an infectious disease physician, who observed lesions compatible with VACV. At the public health clinical evaluation on March 12, the patient had grouping of vesicular lesions with central umbilication on mildly erythematous bases on his trunk (Figure 2), with a solitary lesion near his left areola and on the volar aspect of his right forearm. VACV was clinically diagnosed.

### Case-Patient 4

On March 9, lesions developed along the mandible of the 29-year-old household contact of case-patient 1. On March 11, she was evaluated by a dermatologist, who performed a punch biopsy for suspected herpes. Over the next several days, fever, chills, arthralgias, and submandibular swelling developed. She was seen by the NYSDOH public health physician on March 16, and VACV was clinically diagnosed. The most plausible route of exposure was a shared hand towel with case-patient 1. Over the next several days, her lesions started draining and became substantially more erythematous and painful. She reported a lesion in her right nostril on March 18. After consultation between her physician, NYSDOH, and CDC, varicella immune globulin was released from the Strategic National Stockpile for administration because of the new mucosal involvement (*5*). Varicella immune globulin was administered with no complications on March 19 at a local hospital. The patient reported that the pain associated with her lesions resolved over 24 hours. Her nostril lesion resolved on March 22 with the loss of a small scab.

All 4 cases were clinically diagnosed vaccinia. Samples were obtained by unroofing vesicles, collecting the tissue, performing slide touch preps of the unearthed base of each vesicle, and obtaining viral swabs by using pox collection kits that had been distributed through NYSDOH. All patient specimens were tested at the NYSDOH Wadsworth Center (Albany, NY, USA) by real-time PCR, and preliminary results indicated VACV.

VACV subsequently was confirmed in all 4 patients by real-time PCR at the NYSDOH Wadsworth Center. The complete hemagglutinin gene was sequenced by CDC for 3 of the samples (cases 1, 3, 4) and was identical to that of ACAM2000 (*6*).

NYSDOH provided appropriate transmission precautions and wound care instructions to all 4 case-patients (*7*). Follow-up continued until lesion resolution; no additional VACV cases were identified. Vaccine Adverse Event Reporting System reports were submitted for each case.

## Conclusions

In 2003, members of the military, selected health care workers, public health personnel, and first responders began receiving smallpox vaccinations as part of bioterrorism preparedness (*8*). Although smallpox vaccination campaigns directed toward health care workers and public health officials ended in January 2008 (*9*), smallpox vaccinations continue for military service members. This case report illustrates the need to ensure that military vaccinees understand the risk associated with contact transmission. Regions with active military smallpox vaccination programs need to maintain awareness among community medical providers, health departments, and laboratories to facilitate recognition, correct diagnosis, and appropriate response to inadvertent inoculation of vaccinia virus to help limit further transmission. Especially in areas with ongoing smallpox vaccination programs, appropriate materials for the collection of specimens need to be maintained. Finally, updates are needed on identification of VACV cases, along with the notification and involvement of public health.
